# Significance of ypTNM stage in determining the prognosis and therapy after surgery for locally advanced rectal cancer

**DOI:** 10.1186/s12957-023-03059-w

**Published:** 2023-06-07

**Authors:** Yue Chen, Jiayu Sun, Xinxin Dong, Deyu Sun, Yanli Qu

**Affiliations:** 1 Department of Colorectal Surgery, Cancer Hospital of China Medical University, Liaoning Cancer Hospital & Institute, No. 44 Xiaoheyan Road, Dadong District, Shenyang, Liaoning Province 110042 China; 2 Department of Colorectal Surgery, Department of Internal-Medicine, Health Company of Artillery Brigade Service Support Battalion of the 78Th Group Army of the Northern Theater of Chinese People’s Liberation Army, No.999 Mail Box, Beishan Road, Siping Economic Development Zone, Tiedong District, Siping City, Jilin Province 136001 China; 3 Department of Radiation Oncology Gastrointestinal and Urinary and Musculoskeletal Cancer, Cancer Hospital of China Medical University, Liaoning Cancer Hospital & Institute, No. 44 Xiaoheyan Road, Dadong District, Shenyang, Liaoning Province 110042 China; 4 Department of Abdominal and Lymphoma Radiotherapy, Cancer Hospital of China Medical University, Liaoning Cancer Hospital & Institute, No. 44 Xiaoheyan Road, Dadong District, Shenyang, Liaoning Province 110042 China

**Keywords:** Rectal cancer, Neoadjuvant pathologic TNM stage, Clinical TNM stage, Prognosis, Adjuvant chemotherapy

## Abstract

**Background:**

In the current NCCN guidelines, the prognosis and adjuvant chemotherapy of patients who underwent neoadjuvant chemoradiotherapy (nCRT) are based on pre-radiotherapy clinical TNM (cTNM) stage. However, the value of neoadjuvant pathologic TNM (ypTNM) stage is not clearly described.

**Methods:**

This retrospective study investigated the prognosis and adjuvant chemotherapy which based on ypTNM stage compared to cTNM stage. Between 2010 and 2015, a total of 316 rectal cancer patients who underwent nCRT, followed by total mesorectal excision (TME), were included for analysis.

**Results:**

Our findings revealed that cTNM stage was the only significant independent factor in the pCR group (HR = 6.917, 95% CI: 1.133–42.216, *P* = 0.038). In the non-pCR group, ypTNM stage was more important than cTNM stage in prognosis (HR = 2.704, 95% CI: 1.811–4.038, *P* < 0.001). In ypTNM III stage group, there was a statistically significant difference in prognosis between the patients with and without adjuvant chemotherapy (HR = 1.943, 95% CI: 1.015–3.722, *P* = 0.040), but there was no significant difference in cTNM III stage group (HR = 1.430, 95% CI: 0.728–2.806, *P* = 0.294).

**Conclusions:**

We concluded that ypTNM stage, rather than cTNM stage, might be a more important factor in the prognosis and adjuvant chemotherapy of patients with rectal cancer who underwent nCRT.

## Background

The worldwide incidence of colorectal cancer (CRC) is high [1]. In recent years, the prevalence of CRC has been increasing in part because of an aging population. Among the different types of CRC, nearly 50% are rectal cancers [2]. Many studies have demonstrated that neoadjuvant chemoradiotherapy (nCRT) is effective in reducing local recurrence and preserving the anal sphincter [3, 4]. Therefore, nCRT has become the standard treatment for locally advanced rectal cancer, according to National Comprehensive Cancer Network (NCCN) guidelines [5]. However, the response to nCRT varies from pathological complete response (pCR) to disease progression. According to previous studies, approximately 30% of rectal cancer patients who underwent nCRT showed complete response and approximately 60% showed tumor size regression and N stage descension [6–9]. Due to individual differences in response to nCRT, the tumor stage of patients might vary greatly. In the current NCCN guidelines, the prognosis and adjuvant chemotherapy of patients with nCRT are based on pre-radiotherapy clinical TNM (cTNM) stage. However, the value of neoadjuvant pathologic TNM (ypTNM) stage is not clearly described. Therefore, the aim of this study was to investigate cTNM and ypTNM stages, which was a more important factor in the prognosis and adjuvant chemotherapy of patients with rectal cancer.

## Methods

### Patients

Between 2010 and 2015, we retrospectively analyzed 316 rectal cancer patients who received nCRT, followed by total mesorectal excision (TME) at the Liaoning Cancer Hospital and Institute. Before nCRT, all patients were histologically confirmed to have resectable rectal cancer of clinical T2-4aN0-2M0 stage, according to the 8^th^ edition of the UICC/AJCC TNM classification system. Histological specimens for ypTNM stage were evaluated by two senior pathologists. Patients were excluded if they were diagnosed with unresectable cancer after nCRT, underwent nCRT at other hospitals, died during the peri-operative period, or had incomplete records.

All patients were followed-up for more than 5 years after the surgery. The preoperative staging evaluation included physical and laboratory examinations, enteroscopy with endoscopic ultrasound and pathological biopsy, chest and abdominal computed tomography (CT), and pelvic magnetic resonance imaging (MRI). Most patients were discussed by a multidisciplinary team (MDT) before starting treatment. This study was approved by the Ethics Committee of the Liaoning Cancer Hospital & Institute (NO: 202,204,117) and in accordance with its relevant guidelines and regulations.

### Treatment

All patients were treated with intensity-modulated radiation therapy (IMRT) and volumetric-modulated arc therapy (VMAT) with a minimal photon energy of 6 MV. As for standard dose, after 45 Gy a tumor bed boost with a 2 cm margin of 5.4 Gy in 3 fractions could be considered. Concurrent chemotherapy consisted oral capecitabine (825 mg/m^2^/d twice daily, 5 days a week) during the five weeks of radiotherapy. The mFOLFOX regimen was followed for 1–2 cycles of consolidation chemotherapy during the interval period after the chemoradiotherapy. TME would be performed 2–4 weeks after the end of consolidation chemotherapy. In general, the patients underwent TME after 6 to 8 weeks of nCRT. During the surgery, we tried to preserve the left colon vessels and made a preventive stoma as much as possible. According to NCCN guidelines, patients with cTNM III stage should be treated with mFOLFOX (oxaliplatin + 5-FU + calcium folinate) as adjuvant chemotherapy. However, due to various reasons, some patients did not receive chemotherapy. A pCR was defined as the absence of residual tumor in the entire rectal wall and local lymph nodes. Non-pCR was defined as the presence of residual tumor, either in the rectal wall or local lymph nodes.

### Follow-up

All patients were followed up by telephone interviews or outpatient visits. Patients were followed-up every 3 to 6 months in the first two years and then once per year. At each follow-up, tests included anal examinations, tumor marker levels, abdomen and lung CT, and/or MRI and colonoscopy, if needed. The primary endpoint was overall survival (OS). OS was defined from the day of the surgery to the death of the patient for any reason.

### Statistical analysis

SPSS 22.0 software (IBM, Armonk, NY, USA) was used for statistical analysis. The χ2 test or Fisher’s exact test was used to compare categorical variables. The Kaplan–Meier method was used to assess OS. The Cox proportional hazards regression model was used in forward stepwise multivariate survival analysis. To investigate which TNM stage (cTNM or ypTNM stage) was more important in predicting the prognosis, two-step multivariate survival analysis was used. In step 1 multivariate analysis, all statistically significant prognostic factors from the univariate analysis were included, except for ypTNM stage. In step 2 multivariate analysis, ypTNM stage was also considered, together with statistically significant prognostic factor in step 1. *P* < 0.05 was considered statistically significance.

## Results

### Patient characteristics and follow-up

Patient particulars and clinicopathological characteristics are provided in Table 1. A total of 316 patients with rectal cancer who met the criteria were included in the analysis. The median age was 58 years (range, 16–84 years). All patients underwent TME after nCRT. After histopathological examination, 70 patients (22.2%) achieved pCR, and the remaining patients with residual cancer were classified as the non-pCR group (*n* = 246). Patients with smaller primary tumors, exophytic type cancer and lower cTNM stage were more likely to achieve pCR after nCRT (*P* < 0.001 for all) (Table 1).

**Table 1:** Clinicopathological characteristics of patients with rectal cancer treated with neoadjuvant chemoradiotherapy (*n* = 316)

Clinicopathologic Characteristics	Total (*n* = 316)	pCR (*n* = 70), %	Non-pCR (*n* = 246), %	*P*-value
Age (yrs)				0.466
< 60	173	41(23.7%)	132(76.3%)	
≥ 60	143	29(20.3%)	114(79.7%)	
Sex				0.056
Male	219	42(19.2%)	177(80.8%)	
Female	97	28(28.9%)	69(71.1%)	
Primary tumor Diameter				< 0.001*
< 3 cm	170	53(31.2%)	117(68.8%)	
≥ 3 cm	146	17(11.6%)	129(88.4%)	
Macroscopic type				< 0.001*
Exophytic type	65	41(63.1%)	24(36.9%)	
Ulcerative type	251	29(11.6%)	222(88.4%)	
Histological differentiation				0.726
Well to moderately	276	62(22.4%)	214(77.6%)	
Poorly	40	8(20.0%)	32(80.0%)	
Clinical T stage				0.434
T2	79	20	59	
T3-T4	237	50	187	
Clinical TNM stage				< 0.001*
II	153	51(24.4%)	102(75.6%)	
III	163	19(11.7%)	144(88.3%)	

^*^statistical significance

**Fig. 1:**
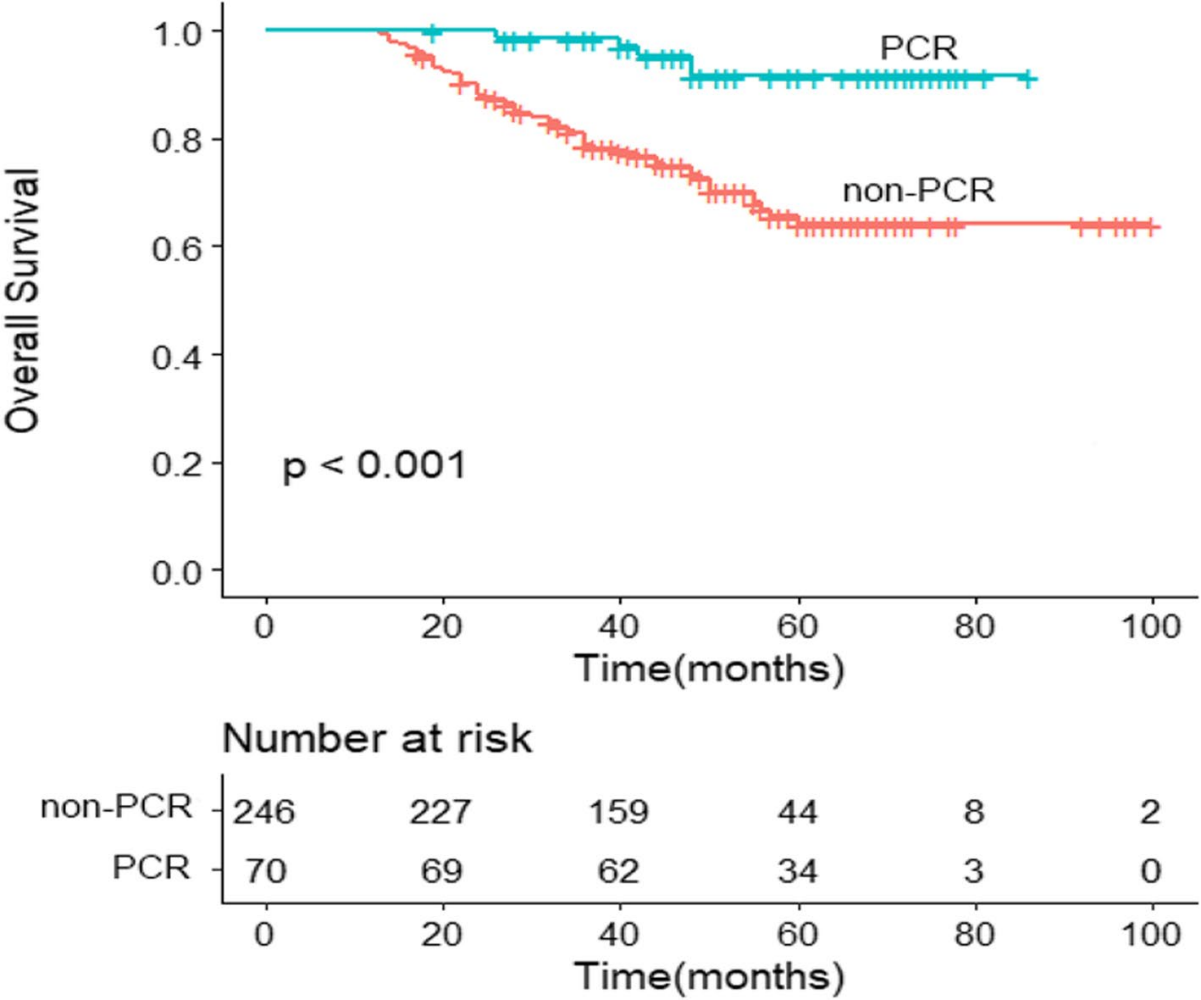
Prognostic analysis of pCR and non-pCR group. There was significant difference between two groups (*P* < 0.001)

The median follow-up time was 47 months (range, 12–101 months) for the 316 rectal cancer patients. At the time of the last follow-up, 73 patients (23.1%) had died due to tumor progression and all patients experienced recurrence: five patients (1.6%) in the pCR group, including 2 cases of local recurrence and 3 cases of distant recurrence, and 68 patients (21.5%) in the non-pCR group, including 12 cases of local recurrence, 42 cases of distant recurrence and 14 cases of concurrent recurrence. One patient died due to an accident. The 5-year OS was 91.5% in the pCR group and 64.1% in the non-pCR group. The OS of the pCR group was better than that of the non-pCR group (HR = 5.083, 95% CI: 2.047–12.627, *P* < 0.001) (Fig. 1).

### Prognostic features of nCRT patients

Univariate and/or multivariate analyses of the prognostic factors in pCR and non-pCR groups are provided in Tables 2 and 3, respectively. Univariate analyses showed that primary tumor diameter (*P* = 0.023) and cTNM stage (*P* = 0.015) entered into multivariate analysis in the pCR group. Moreover, multivariate analysis demonstrated that cTNM stage was the only significant independent factor (HR = 6.917, 95% CI: 1.133–42.216, *P* = 0.038). For the non-pCR group, histologic differentiation (*P* = 0.052), cTNM stage (*P* = 0.021), and ypTNM stage (*P* < 0.001) were associated with the prognosis of patients who underwent nCRT. To determine which factor (histologic differentiation, cTNM stage, or ypTNM stage) was the most important in predicting the prognosis, two-step multivariate analysis was applied (Table 4). In step 1, the significant factors (histologic grade and cTNM stage) from the univariate analysis were considered, except for ypTNM stage, and cTNM stage was confirmed to be an independent factor in predicting a better prognosis (HR = 1.811, 95% CI: 1.084–3.025, *P* = 0.023). In step 2, when ypTNM stage was considered, ypTNM stage rather than cTNM stage became the most important prognostic factor (HR = 2.704, 95% CI: 1.811–4.038, *P* < 0.001). In other words, ypTNM stage was a more important prognostic factor than cTNM stage.

**Table 2:** Univariate and multivariate prognostic analysis for pCR patients with colorectal cancer (*n* = 70)

Clinicopathologic Characteristics	n	Univariate			Multivariate
		5-year overall survival rate	Hazard ratio (95% CI)	*P*-value	*P*-value
Age (yrs)			1.008(0.168–6.048)	0.993	
60	41	91.7%			
≥ 60	29	91.2%			
Sex			0.358(0.040–3.205)	0.335	
Male	42	89.0%			
Female	28	95.2%			
Primary tumor Diameter			6.138(1.022–36.869)	0.023*	0.076
< 3 cm	53	95.2%			
≥ 3 cm	17	78.7%			
Macroscopic type			2.032(0.340–12.163)	0.426	
Exophytic type	41	93.1%			
Ulcerative type	29	89.0%			
Histological differentiation			1.796(0.200–16.116)	0.594	
Well to moderately	8	92.5%			
Poorly	62	85.7%			
Clinical T stage			0.323(0.054–1.938)	0.191	
T2	20	84.4%			
T3-T4	50	95.1%			
Clinical TNM stage			6.917(1.133–42.216)	0.015*	0.038*
II	51	95.6%			
III	19	74.7%			

^*^statistical significance

**Table 3:** Univariate prognostic analysis for non-pCR patients with colorectal cancer (*n* = 246)

Clinicopathologic Features	n	5-year overall survival rate	Hazard ratio (95% CI)	*P*-value
Age (yrs)			0.714 (0.438–1.162)	0.171
< 60	132	60.5%		
≥ 60	114	69.0%		
Sex			0.943 (0.556–1.599)	0.826
Male	177	61.5%		
Female	69	69.8%		
Primary tumor Diameter			1.254 (0.779–2.020)	0.348
< 3 cm	117	70.1%		
≥ 3 cm	129	57.4%		
Macroscopic type			1.406 (0.607–3.259)	0.422
Exophytic type	222	68.8%		
Ulcerative type	24	64.2%		
Histological differentiation			1.771 (0.985–3.186)	0.052
Well to moderately	214	67.3%		
Poorly	32	45.0%		
Clinical TNM stage			1.811 (1.084–3.025)	0.021*
II	102	71.2%		
III	144	59.0%		
Neoadjuvant pathologic TNM stage			2.704 (1.811–4.038)	< 0.001*
I	51	96.0%		
II	94	66.4%		
III	101	49.7%		

^*^statistical significance

**Table 4:** Two-step multivariate analysis of the prognostic factors for non-pCR patients with colorectal cancer

	Hazard ratio	95% CI	P
Step 1			
Histological differentiation			0.069
Clinical TNM stage	1.811	1.084–3.025	0.023*
Step 2			
Clinical TNM stage			0.974
Neoadjuvant pathologic TNM stage	2.704	1.811–4.038	< 0.001*

Step 1, with consideration of all significantly important prognostic factors in univariate analysis except for neoadjuvant pathologic TNM stage after surgery

Step 2, with consideration of clinical TNM stage and neoadjuvant pathologic TNM stage in univariate analysis

^*^statistical significance

### Adjuvant chemotherapy for nCRT patients

Due to adjuvant chemotherapy is mostly performed in patients with TNM III stage, we performed prognostic analysis in cTNM III and ypTNM III stage group, respectively.

**Fig. 2:**
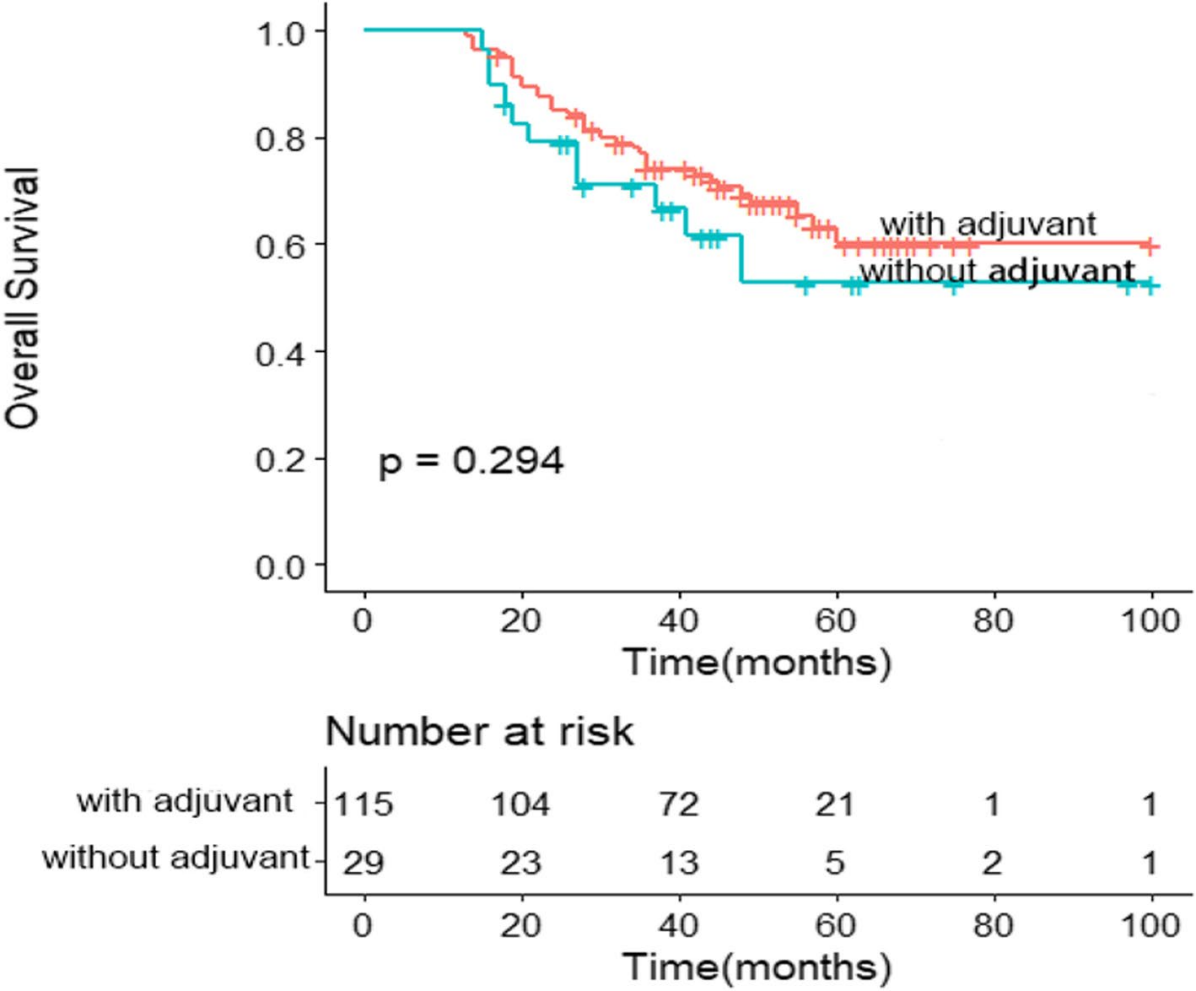
Prognostic analysis of patients with adjuvant chemotherapy and without adjuvant chemotherapy in cTNM III stage group. Patients with adjuvant chemotherapy has a trend of better prognosis than without adjuvant chemotherapy, but there is no significant statistical difference (*P* = 0.294)

**Fig. 3:**
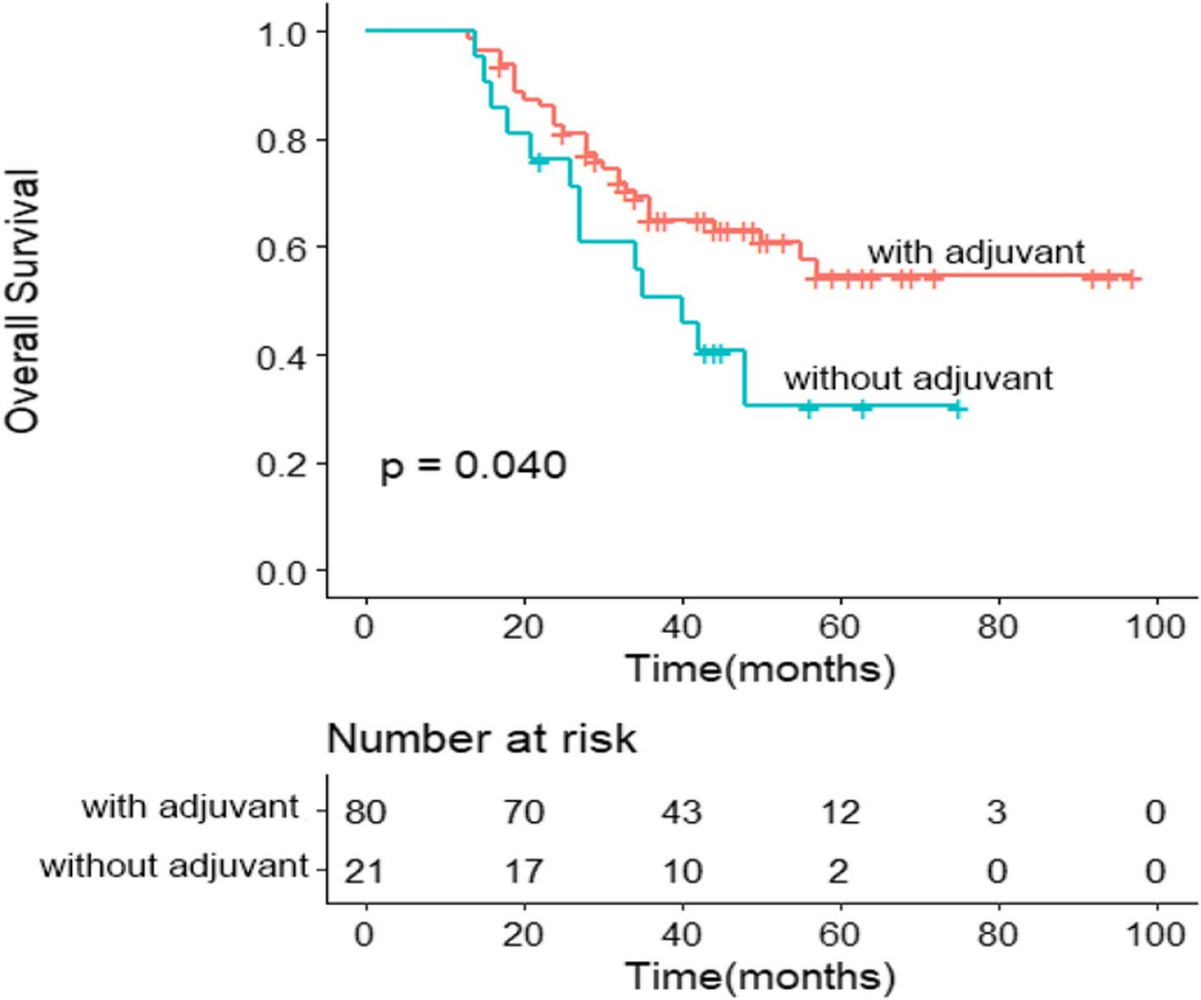
Prognostic analysis of patients with adjuvant chemotherapy and without adjuvant chemotherapy in ypTNM III stage group. Patients with adjuvant chemotherapy has a better prognosis than without adjuvant chemotherapy (*P* = 0.040)

In cTNM III stage group, 115 patients (79.9%) received adjuvant chemotherapy and 29 patients (20.1%) received no chemotherapy, including 18 patients with ypTNM III stage. Patients with adjuvant chemotherapy had a trend of better prognosis than without adjuvant chemotherapy with 5-year OS rates of 60.1% versus 52.8%, but there was no significant statistical difference (HR = 1.430, 95% CI: 0.728–2.806, *P* = 0.294) (Fig. 2). Furthermore, in ypTNM III stage group, 5-year OS rates of patients with adjuvant chemotherapy had better than patients without adjuvant chemotherapy (54.6% versus 30.5%, and HR = 1.943, 95% CI: 1.015–3.722, *P* = 0.040) (Fig. 3).

## Discussion

According to the current NCCN guidelines, the adjuvant treatment and prognosis of rectal cancer after surgery are based on pre-radiotherapy cTNM stage [10]. In this study, we found that ypTNM stage might be a more accurate factor to reflect the prognosis and guiding adjuvant therapy of rectal cancer patients who underwent nCRT. Many studies have also reported the importance of the neoadjuvant pathological stage in the prognosis of patients. Sun et al. [11] investigated 317 rectal cancer patients who underwent radical surgical resection following nCRT and observed that ypTNM stage was the only independent risk factor in these patients. Similarly, Kim et al. [12] reported that ypTNM stage was an important prognostic factor in the prediction of local recurrence and distant metastasis in rectal cancer patients. Since the TNM stage of patients might vary greatly after nCRT, there might be significant variations in prognosis based on cTNM stage. Therefore, we concluded that ypTNM stage might better reflect the prognosis of patients than cTNM stage.

NCCN guidelines recommend adjuvant treatment for patients with cT3-4N0 and cT1-3N1-2 stage after nCRT. Is it suitable? We all known that the response to nCRT varies from pCR to disease progression due to individual differences in response to nCRT. Whether ypTNM stage after nCRT is more accurate in guiding adjuvant therapy than cTNM stage is worth studying. A multicenter randomized controlled clinical study confirmed that oxaliplatin + 5-FU combination chemotherapy can significantly improve the 3-year disease-free survival of patients with ypTNM stage III rectal cancer compared with 5-FU chemotherapy alone, but it had no effect on the prognosis of patients with ypTNM stage II rectal cancer [13]. You et al. [14] performed a retrospective study of 160 rectal cancer patients and observed that adjuvant chemotherapy might not improve the survival of ypT0-2N0 patients but might be meaningful for ypT3-4N0 patients in terms of the 5-year OS. These studies indicated that ypTNM stage had important value in guiding adjuvant therapy. In our study, the patients with adjuvant chemotherapy had a better prognosis than the patients without adjuvant chemotherapy in ypTNM III stage group, but there was no statistical difference in cTNM III stage. Therefore, we concluded that adjuvant therapy based on ypTNM stage might be more accurate.

The main advantage of our study was that we investigate the value of ypTNM stage from both treatment and prognosis. However, there were several limitations in the current study. First, the sample size was relatively small, which contributed to the low statistical power of the prognostic comparisons. Second, because of the nature of retrospective studies, selectivity bias was inevitable. Therefore, further studies should be carried out to confirm our results.

In conclusion, our study showed that ypTNM stage might be a more accurate factor to reflect the prognosis and guiding adjuvant therapy of patients with rectal cancer who underwent nCRT, which was not clearly pointed out in the current NCCN guidelines.

This study provides evidence of more accurate therapy and prognosis after nCRT. For further research, we are currently conducting a large multicenter retrospective study.

## Data Availability

Not applicable.

## Abbreviations

nCRT: Neoadjuvant chemoradiotherapy
cTNM: Clinical TNM
ypTNM: Neoadjuvant pathologic TNM
CRC: Colorectal cancer
NCCN: National Comprehensive Cancer Network
pCR: Pathological complete response
TME: Total mesorectal excision
CT: Computed tomography
MRI: Magnetic resonance imaging
MDT: Multidisciplinary team
IMRT: Intensity-modulated Radiation Therapy
VMAT: Volumetric-modulated Arc Therapy
OS: Overall survival
